# Comparison of albumin and cabergoline in the prevention of ovarian hyperstimulation syndrome: A clinical trial study

**Published:** 2013-10

**Authors:** Aalie Torabizadeh, Fatemeh Vahidroodsari, Zakieh Ghorbanpour

**Affiliations:** 1 *Department of Obstetrics and Gynecology, Ghaem Hospital, Mashhad University of Medical Sciences, Mashhad, Iran. *; 2 *Ghaem Hospital, Mashhad University of Medical Sciences, Mashhad, Iran. *

**Keywords:** *Ovarianhyperstimulationsyndrome (OHSS)*, *Cabergolin*, *Assistedreproductivetechnology(ART)*, *Albumin*

## Abstract

**Background:** Ovarian hyperstimulation syndrome (OHSS) is the most serious and potentially life-threatening iatrogenic complication associated with ovarian stimulation during Assisted Reproductive Technology (ART) protocols. OHSS typically is a result of ovarian expression of vascular endothelial growth factor (VEGF) which increases vascular permeability.

**Objective:** Comparison of albumin and cabergoline in the prevention of OHSS.

**Materials and Methods:** 95 high risk infertile women for OHSS (more than 20 follicles in both ovaries at day of Human Chorionic Gonadotropin (HCG) injection) were randomly divided into two groups. First group including 48 women received 10 unit intravenous albumin at starting oocyte retrieval, and second group including 47 women received 0.5 mg/day dopamine agonist (Cabergolin) at day of HCG injection till 8 days. The dosage of human Menopausal Gonadotropin (HMG) used, total number of follicles developed, number of oocytes retrieved, serum E_2_ concentrations during the luteal phase, development of ascites, number of embryos generated, clinical pregnancy rate, results of the in vitro fertilization-embryo transfer (IVF-ET) cycles and incidence and severity of any OHSS were evaluated.

**Results:** There was evidence of a statistically significant reduction in the incidence of OHSS in the cabergolin group (53.7%) versus albumin group (46.3%) (p=0.04). But there was no significant difference of a reduction in severe OHSS (p=0.62). There was no difference in clinical pregnancy rate too.

**Conclusion:** Administration of cabergolin can prevent incidence of OHSS and does not appear to effect on its severity.

Registration ID in IRCT: IRCT138706281217N4

## Introduction

Ovarian hyperstimulation syndrome (OHSS) is a serious iatrogenic complication of ovulation induction and ovarian stimulation for assisted reproductive technology (ART). Although significant OHSS has a relatively low incidence (2.1%), it may in severe cases result in a potentially life-threatening situation (1). Exogenous or endogenous HCG is the triggering factor of this syndrome. The relationship between HCG and OHSS is thought to be production of the vascular endothelial growth factor (VEGF) (2, 3). The pathophysiology of OHSS is still not well understood, but different factors related to an increased capillary permeability have been involved, leading to a wide and varied spectrum of clinical presentation. The intensity of the syndrome is related to the degree of the follicular response in the ovaries to the ovulation-inducing agents. This classification categorizes patients according to mild, moderate and severe disease (4, 5).

In mild OHSS patients report mild abdominal distention, nausea and vomiting ovarian enlargement can be 5-12 cm. Moderate disease is marked by presence of abdominal ascites on ultrasound exam. Severe OHSS is characterized by a tense ascites, hydrothorax, hemoconcentration, hypercoagulability or any complication of OHSS such as renal failure, thromboembolism and acute respiratory distress syndrome (ARDS) (6). Patients should be hospitalized in moderate and sever cases. There is no specific therapy for OHSS and treatment is conservative. The prevention of OHSS is very important and should be considerate (7).

Suitable primary predictors and tests which to identify susceptible patients are remained unreliable. Although anti-mullerian hormone and antral follicle count are currently suitable risk factors candidates as primary prevention (8-10). Secondary preventions include canceling of cycle, coasting, the use of antagonist protocol and trigger of last oocyte maturation with gonadotropin releasing hormone (GnRH) agonist crayon preservation of all embryos and in vitro maturation of oocyte (IVM) (11-15). Recently, vascular endothelial growth factor (VEGF) has been identified for vascular permeability that is associated with OHSS (16). 

Usage of dopamine agonist, cabergoline has been found to reduce the effects of VEGF without compromising pregnancy rate (17). Administration of intravenous albumin at time of oocyte retrieval has been studied as a possible prevention strategy (18). Albumin seems to have osmotic functions, as it contributes to around 75% of the plasma oncotic pressure, drawing extracellular fluid into the circulation, and possesses transport functions, binding and inactivating the vasoactive intermediates responsible for the pathogenesis of OHSS (19). 

A meta-analysis from the Cochrane database includes five trials of 378 patients deemed to be at high risk for severe OHSS. The treatment regimens varied from (10-50 gr) of albumin given one or two hours before oocyte retrieval. Overall severe OHSS developed in 14 of 185 patients treated with placebo compared with albumin 4 of 193. Four of five trials showed a benefit. But one study did not achieve same results (20). Data about the efficacy of IV albumin administration for OHSS prevention are conflicting (21). In this study we compared the effect of cabergolin and albumin in the prevention of OHSS and its severity. 

## Materials and methods

In this clinical trial study, infertile women referred to Montasareye Infertility Center, Mashhad, Iran were evaluated in 2009. The inclusion criteria were the presence of >20 oocytes during oocyte retrieval, ovary size >10 cm, serum estradiol >2500 pg/ml on the day of HCG administration. The study protocol was approved by the Mashhad University of Medical Sciences Ethical Committee. All participating patients provided written informed consent at their first visit. Each participant underwent a complete evaluation including clinical history, physical and ultrasound examination and hormonal profile. 

The sample volume was calculated according to the study of Ben-Chetrit *et al* that in their study, the severity of OHSS in albumin group was 0.78 and in control group 0.92 (21). The method of sampling was randomized sampling as we selected every other person. The physician who has controlled the patients was blind. The patients were down regulated to the long protocol (50 IU, Superfact; Aventis Pharma Deutshlan, Frankfurt, Germany) with gonadotropins such as HMG, purified urinary follicular stimulating hormone (Fostimone), synthetic follicle stimulating hormone (FSH) (Gonal-F; Serono Laboratories Ltd., Geneva, Switzerland) and HCG to stimulate the ovaries.

**Figure 1:**
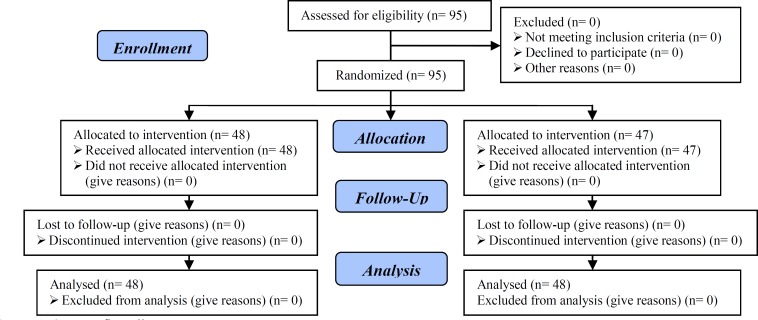
Consort flow diagram

Patients were recurrently followed by transvaginal ultrasonography (TVS) to evaluate number and size of the ovarian follicles. 95 high risk patients for OHSS (more than 20 follicles in both ovaries at day of HCG injection) were randomly divided into two groups (Figure 1). Randomization was used to allocate the patients to two groups immediately after confirmation of retrieval of >20 oocytes. First group including 48 patients received 10 units intravenous albumin (Albúmina humana Grifols 20%; Grifols, Barcelona, Spain) at starting oocyte retrieval, and second group including 47 patients received 0.5 mg/day dopamine agonist (cabergolin) (0.5 mg/day by mouth; Dostinex; Pfizer Italia S.r.l., Ascoli Piceno, Italy) at day of HCG injection till 8 days. 

Monitoring was done by clinical symptoms, signs and lab data such as createnine, PTT, PT, electrolytes, CBC. The patients who were complicated with moderate or severe OHSS admitted in hospital and followed up. β-hCG was checked 16 days after embryos transfer. Randomization was strictly followed over the study period. Patients who were admitted classified in sever and moderate OHSS with clinical and laboratory symptoms in 2 groups were matched together according to the duration of infertility, etiology of infertility, numbers of follicles at day of hCG injection.

The incidence in the studied groups (albumin vs. cabergolin) of moderate and severe OHSS and biochemical serum changes were the primary outcome measures. The implantation and pregnancy rates in patients were the secondary outcome measures. 

In both groups, hematological tests performed immediately following oocyte retrieval and again 7 days later. CBC diff, Na, K, Urea, Hemoglobin, hematocrit, leukocyte count, platelet count, PT, PTT, renal (creatinine) and liver [transaminases: aspartate aminotransferase (AST); alanine aminotransferase (ALT)] functions were analyzed. Women were monitored on a non‐rigid outpatient basis via phone contact and visits until menstruation occurred or until fetal heart activity was detected in pregnant patients. Cases of OHSS were classified according to related criteria. 

**Statistical analysis**

To describe quantitative data (demographic characteristics) the tables were used. Chi-square test and student t-test were used for data analyzing. If there was no condition for performing parametric tests, nonparametric Mann-Whitney test was employed. Data analysis was performed by SPSS version 16 and p≤0.05 was considered statistically significant. 

## Results

In this study, no significant differences regarding infertility duration and type infertility and numbers of follicles on day of HCG injection (p=0.6). 

13 of 95 patients were admitted in hospital because of severe and moderate OHSS symptoms (13%). From these patients 10 women were received albumin (76.8%) and 3 women were received cabergolin (23%), that there was definitive decrease in OHSS incidence in cabergolin group vs. albumin (p=0.04) (Table I). Admitted women were compared together regarding pregnancy and was no difference between them (p=0.6) (Table II). 

Moderate OHSS was observed 5 cases in Albumin group and 2 cases in cabergolin group. Sever OHSS was observed 5 cases in Albumin group and 1 cases in cabergolin group. Which were not any significant differences between two groups (p=0.6) (Table III).

**Table I:** OHSS incidence in cabergolin group vs. albumin group (p=0.04)

**Type of drug**	**Ovarian hyperstimulation syndrome**		**Total**
	**No**	**Yes**	
Albumin	38 (46.3%)	10 (76.9%)	48 (50.5%)
Cabergoline	44 (53.7%)	3 (23.1%)	47 (79.5%)
Total	82 (100%)	13 (100%)	95 (100%)

Numbers are presented as [n (%)]. Student t-test (p=0.04)

**Table II:** Percent of Pregnancy in cabergolin group vs. albumin group

**Pregnancy**	**Drugs**	
	**Albumin**	**Cabergolin**
No	5 (50%)	1 (33.3%)
Yes	5 (50%)	2 (66.7%)

Numbers are presented as [n (%)]. Mann-Whitney test (p=0.612).

**Table III:** OHSS severity in Cabergolin group vs. albumin group

**OHSS**	**Drugs**		**Total**
	**Albumin**	**Cabergolin**	
Moderate	5 (50%)	2 (66.7%)	7 (53.8%)
Severe	5 (50%)	1 (33.3%)	6 (46.2%)
Total	10 (100%)	3 (100%)	13 (100%)

Numbers are presented as [n (%)]. Mann-Whitney test (p=0.6).

## Discussion

In this study we compared IV Human Albumin with Dopamine agonist, cabergolin in infertile patient at high-risk for OHSS in IVF\ICSI cycles. We identified a significantly decrease in OHSS incidence in cabergolin group vs. albumin. The study of Youssef *et al* in 2010 on four randomized trials (n=570 women) showed a statistically significant reduction in the incidence of OHSS in the cabergolin group versus no treatment group (22). Carizz *et al* compared IV albumin users and cabergolin users. They found that although the risk of early OHSS was decreased significantly in cabergolin group but the risk of late onset OHSS did not change (23). In a meta-analysis, 988 women undergoing IVF were randomly assigned to IV albumin or no treatment on day of oocyte retrieval that demonstrated no beneficial effect of albumin therapy (24). 

Tehraninejad *et al* performed a study to compare the efficacy of cabergoline (Cb2) and intravenous human albumin (HA) in the prevention of OHSS and reported that prophylactic oral low dose cabergoline was more effective and less costly than intravenous human albumin in the prevention of OHSS in high-risk patients (25). Rollen *et al* in 2009, found when dopamine agonist with GnRH antagonist protocol given together at the time of OHSS diagnosis, clinical symptom of the disease diminished rapidly and OHSS severity suppressed effectively (26). In this study patients received 0.5 mg oral cabergolin daily for 21 days beginning on the day after oocyte retrieval but we prescribed to our patients 0.5 mg per day just for 8 days from HCG injection as preventive agent. 

Shaltout *et al* reported that the overall incidence of OHSS was significantly reduced, almost 50%, in cabergoline group in comparison with control group (PR: 0.5, 95% CI: 0.29-0.83), with absolute risk reduction following cabergoline administration 11% (27). Ata and coworkers found when cabergolin dose was increased to 1 mg per day after oocyte collection can treat and resolve OHSS symptoms (28). In our study there was no statistically significant evidence of a reduction in severe OHSS between two groups. Youssef and Carizzac also found no reduction in severe OHSS in cabergolin users (22, 23). Saylan *et al* showed low dose cabergolin was ineffective in severe OHSS (29). In this study there was no evidence for a difference in clinical pregnancy rate in hospitalized patient for moderate and severe OHSS in two groups. Other studies also presented no changes in pregnancy and miscarriages rates (23-24). 

## Conclusion

In conclusion, oral administration of cabergolin decreases the incidence of OHSS than albumin. Moreover, cabergolin is most cost-effective and safer than IM administration of albumin. However, further studies about the best time and dose for the drug administration are needed.
